# The disruptive – and beneficial – effects of distraction on older adults’ cognitive performance

**DOI:** 10.3389/fpsyg.2014.00133

**Published:** 2014-02-18

**Authors:** Jennifer C. Weeks, Lynn Hasher

**Affiliations:** ^1^Department of Psychology, University of TorontoToronto, ON, Canada; ^2^Rotman Research Institute, Baycrest CentreToronto, ON, Canada

**Keywords:** aging, attention regulation, distraction, inhibition, facilitation

## Abstract

Older adults’ decreased ability to inhibit irrelevant information makes them especially susceptible to the negative effects of simultaneously occurring distraction. For example, older adults are more likely than young adults to process distraction presented during a task, which can result in delayed response times, decreased reading comprehension, disrupted problem solving, and reduced memory for target information. However, there is also some evidence that the tendency to process distraction can actually facilitate older adults’ performance when the distraction is congruent with the target information. For example, congruent distraction can speed response times, increase reading comprehension, benefit problem solving, and reduce forgetting in older adults. We review data showing that incongruent distraction can harm older adults’ performance, as well as evidence suggesting that congruent distraction can play a supportive role for older adults by facilitating processing of target information. Potential applications of distraction processing are also discussed.

People often prefer to work in quiet, distraction-free environments when doing cognitively demanding tasks such as reading, driving, or solving a puzzle. Quiet typically improves task performance because it allows a person to concentrate their attentional resources on the task at hand (Kahneman, 1973), possibly by minimizing the amount of interference created by irrelevant information (Hasher and Zacks, 1988).

The desire to work in a quiet environment may increase with age as people become even more susceptible to the disruptive effects of distraction (Hasher and Zacks, 1988). This idea is supported by a good deal of laboratory based evidence, from simple response time measures to more complex tasks involving problem solving and reading for comprehension, all showing that irrelevant distraction has an especially negative effect on older adults’ performance.

Contrary to popular belief, however, the consequences of older adults’ tendency to process distraction are not always negative. In this paper, we review evidence that the content of distracting information, specifically its relevance to target information, determines whether it will help or hinder older adults’ performance. Following a brief section on potential neural underpinnings of this phenomenon, we begin with a review of the abundant evidence showing that incongruent distraction is especially disruptive in old age. Next, we turn to the growing literature showing that congruent distraction can actually benefit older adults and, where gaps in the literature exist, we make predictions for future results based on extant evidence. Finally, we suggest some possible ways in which beneficial distraction may help older adults function optimally in the real world.

## NEURAL UNDERPINNINGS OF DISTRACTER PROCESSING

The neural basis for this age-related inhibitory deficit is gradually being revealed through the use of neuroimaging techniques. Functional MRI studies have implicated a widespread network of frontal and parietal brain regions as the basis for top-down attentional control in young adults (Corbetta and Shulman, 2002; Vincent et al., 2008; Spreng et al., 2010). This frontoparietal network, which includes the rostral prefrontal cortex, and inferior parietal cortex, is recruited by young adults when they are told to ignore salient distracters, and its activation is associated with decreased priming for distraction (Campbell et al., 2012). However, connectivity between these regions is reduced in older adults (Madden et al., 2010; Campbell et al., 2012; Li et al., 2012), who also show a corresponding increase in priming for distraction (Campbell et al., 2012). Therefore, a breakdown in the intrinsic connectivity of the frontoparietal control network with age may dysregulate top-down attention (Campbell et al., 2012), resulting in the processing of distracters by older adults. In many scenarios, increased processing of distracters is detrimental to cognitive performance; however, evidence shows that processing non-target stimuli that are congruent with task goals can in fact facilitate perception of target stimuli, leading to enhanced task performance (e.g., May, 1999; Yang and Hasher, 2007; Mozolic et al., 2012).

## WHEN DISTRACTION HARMS

### RESPONSE TIMES

Older adults’ difficulty in ignoring distracting information is perhaps most apparent in their performance on typical tasks of interference control, such as the Stroop (1935) and flanker (Eriksen and Eriksen, 1974) tasks. In these tasks, the critical trials contain distraction that is in direct competition with the required response. Performance on these trials compared to control trials is an index of distracter processing. As expected, older adults show a disproportionate slowing on interference trials compared to younger adults in both the Stroop (Spieler et al., 1996; West and Alain, 2000) and flanker (Zeef et al., 1996) tasks. Older adults are also slowed by distraction on tasks that are not typically thought of as containing interference. For example, Lustig et al. (2006) showed that older adults were faster to indicate whether or not two sets of letters are the same (e.g., RXLTVY_RXLTVY) when only one pair was presented at a time compared to when many pairs were presented simultaneously. It is noteworthy that this manipulation did not affect the response times of younger adults. Interestingly, this result suggests that older adults’ response times may be overestimated in any test that contains visual clutter, due to their reduced ability to filter out irrelevant information (Hasher and Zacks, 1988).

Auditory and even multimodal distraction can also be disruptive to older adults. For example, older adults show larger auditory Stroop (e.g., Sommers and Huff, 2003) and Simon (e.g., Pick and Proctor, 1999) effects than do younger adults. When participants were asked to make a lexical judgment about a spoken word and ignore its tone of voice, older adults were slower to respond to a word that was spoken in an incongruent tone of voice (e.g., “annoyed,” spoken in a happy tone) than a congruent tone of voice (e.g., “annoyed,” spoken in an annoyed tone), but no similar slowing effect was found in younger adults (Wurm et al., 2004). Additionally, older adults may be especially susceptible to distraction presented in a different modality from target information (Guerreiro et al., 2013b). For instance, older adults’ responses on a visual digit categorization task (i.e., “Is this digit odd or even?”) were reported to be disproportionately slowed when trials were preceded by an oddball noise compared to a standard noise with which they were very familiar (Parmentier and Andrés, 2010, but see Guerreiro et al., 2013a).

In light of evidence that older adults are more susceptible than young adults to distraction in multiple modalities, as well as across modalities, it is likely that increased distracter processing reflects an age-related decline in a central inhibitory mechanism (Hasher and Zacks, 1988), rather than a decrease in the integrity of any one sensory system. Further support for this idea comes from a recent finding showing that older adults’ resistance to auditory distraction in a speech-in-noise task can be predicted by their resistance to visual distraction in a Stroop task, above and beyond the predictive effect of hearing loss (Janse, 2012).

### PROBLEM SOLVING

The findings reviewed above suggest that distraction can disrupt older adults’ performance in a wide variety of tasks. One can then ask how much older adults actually know about the irrelevant distraction. Work by May (1999) shed light on this question by showing that semantically misleading distracters can impair older adults’ performance on a problem solving task. In this study, older and younger adults performed the Remote Associates Task (Mednick, 1962), in which they were asked to identify a word that connects three cue words (e.g., SHIP, OUTER, CRAWL; answer: space) while ignoring concurrently presented distracter words. When distracter words were misleading, that is, when they were related to the incorrect interpretation of the cue word [e.g., ocean (SHIP), inner (OUTER), baby (CRAWL)], older adults’ problem solving suffered. Thus, older adults are not just slowed by response-incompatible distraction; they also conceptually process the meaning of distracters and this can impact higher order tasks like problem solving.

### COMPREHENSION AND MEMORY

The tendency to conceptually process distracters also has implications for reading comprehension. There is considerable evidence that older adults have more difficulty reading written passages that are interspersed with visually distinct distracting words, especially when the distracting words are semantic competitors of words in the passage (Connelly et al., 1991; Duchek et al., 1998; Darowski et al., 2008). After reading such passages, older adults are also more likely than younger adults to incorrectly answer comprehension questions with the distracting words (McGinnis, 2012). This finding suggests that irrelevant information processed during reading may distort older adults’ interpretation of text. Although passages with deliberately inserted distracter words are uncommon in the real world, having the television or radio on while reading could influence older adults’ comprehension of text, which might be especially problematic if they are reading information with medical or legal relevance.

Perhaps not surprisingly, distraction likewise influences memory of to-be-learned information. For example, older adults but not younger adults showed reduced free recall of a text when it was interspersed with distracting words compared to when it was not (Mund et al., 2012). In a similar task in the auditory domain, older but not younger adults showed worse recall of spoken sentences masked by meaningful distracter speech compared to spoken sentences masked by random word strings (Tun et al., 2002). In a cross-modal study in which participants memorized written passages while listening to irrelevant distracter speech, older adults made more intrusions that were related to the distracter speech in their recollection of the passages than did younger adults (Bell et al., 2008). Together, these findings suggest that processing irrelevant distraction during encoding, as older adults do, cannot only reduce memory for targets, but also contaminate memory by coloring it with the semantic content of the distraction.

Just as distraction at encoding has an especially deleterious effect on memory for older adults, so does distraction at retrieval. Older adults but not younger adults remembered fewer details about previously studied objects when they were fixating their gaze on an unrelated distracter picture during retrieval than when they were fixating on a gray screen (Wais et al., 2012). Older adults seem to be more susceptible to interference from incongruent distraction at both encoding and retrieval stages of memory (but see Fernandes and Moscovitch, 2003).

In summary, incongruent or irrelevant distraction can be particularly disruptive to older adults’ performance on a wide range of laboratory tasks. The negative effect of distraction on older adults also has real world consequences, given that impaired attentional control in old age has been associated with an increased risk of falls (Mirelman et al., 2012; Amboni et al., 2013), traffic accidents (Nagamatsu et al., 2011; Neider et al., 2011), and driver errors (Hoffman et al., 2005; Thompson et al., 2012).

## WHEN DISTRACTION HELPS

There is substantial evidence, then, that older adults process distraction both perceptually and conceptually, and this tendency frequently impairs their cognitive performance relative to that of younger adults’. There are also findings showing that older adults can actually benefit from the presence of distraction, an effect that can be seen when the distraction is congruent with the task that they are performing. The benefits of distraction processing have received noticeably less empirical attention than have the costs of distraction processing, so, where appropriate, we also identify gaps in the literature and offer our predictions for future work in this area.

### REACTION TIMES

In simple target detection tasks, older adults have been shown to reliably benefit from multisensory targets more than young adults do (Mozolic et al., 2012). Remarkably, older adults’ response times in detecting visual stimuli onset were faster than younger adults’ responses when an auditory tone was played at target onset, even though no age differences in unisensory target response times were seen (Peiffer et al., 2007). In another study, older adults’ saccades toward visual targets were speeded to a greater degree than younger adults’ when a spatially congruent tone was played at target onset, and this was true even in the presence of visual distraction (Campbell et al., 2010).

Perceptual facilitation by distraction can sometimes be seen in older adults’ Stroop performance as well. Spieler et al. (1996) found a numerical but not statistically significant speeding of reaction times on congruent trials compared to no distraction trials in older but not younger adults. Interestingly, the facilitation of response time by congruent distraction was markedly increased in patients with Alzheimer’s disease, which is also characterized by a decrease in executive functions including resistance to distraction (Baddeley et al., 2001). These results suggest that the capture of attention by distraction in older adults happens at a relatively low level, and can benefit target detection in older adults when the distraction is congruent with the required response.

Older adults’ response times can also be speeded by the presence of a distracter that is conceptually congruent with the target. The conceptual congruency between target and distracter should facilitate target processing to the extent that an individual processes the distraction. Yang and Hasher (2007) demonstrated precisely this effect. They measured the time it took younger and older adults to indicate whether two successively presented words were semantically similar, depending on whether the first word was superimposed over a semantically congruent or incongruent picture that was irrelevant to the task. They found that older adults showed a much greater facilitation effect for the congruent pictures than the younger adults did. Therefore, while response times in old age can be slowed by irrelevant distraction, the evidence reviewed here suggests that they can also be speeded by congruent distraction.

### PROBLEM SOLVING

The tendency to conceptually process distraction can also benefit higher order cognition, such as problem solving. In the previously described study by May (1999), older adults’ performance on the Remote Associates Test was shown to be improved in a condition where the distracter word primed the *correct *interpretation of the cue words. For example, for the cue words SHIP, OUTER, CRAWL, the solution is “space.” When distracter words primed the correct interpretation of the words, [e.g., rocket (SHIP), atmosphere (OUTER), or attic (CRAWL)], the older adults were more likely to solve the problem than when the distraction primed the incorrect interpretation of the words, even though they reported not looking at the distracters. In this way, problem solving was enhanced by capitalizing on older adults’ tendency to conceptually process distraction. Interestingly, older adults’ problem solving was also enhanced on the Remote Associates Task when the solution words appeared as distraction in a previous task (Kim et al., 2007), suggesting that older adults retain the semantic content of distraction for some length of time even after the distraction has been removed.

### COMPREHENSION AND MEMORY

If unintentionally processing task-congruent, non-target items can enhance problem solving, then the same might be true for reading comprehension. Surprisingly, given the large number of aging studies that have used the reading with distraction paradigm, the effect of semantically congruent distracters on reading comprehension in this paradigm has yet to be tested. Based on the May (1999) data reviewed above, one would predict that older adults’ reading times and/or comprehension of a written passage may be improved if distracters were synonyms of important words in the passage instead of semantic competitors as in previous studies (e.g., Connelly et al., 1991).

A few studies have tested whether older adults’ reading comprehension is improved by the addition of aids such as illustrative graphics or simultaneous listening while reading. In one such study, Griffin and Wright (2009) asked younger and older adults to read informational leaflets containing embellishing (i.e., non-informative) graphics, explanatory (i.e., conceptually relevant) graphics, or just text and no graphics, and tested the time they took to answer comprehension questions about the material. They found that there was an age-related slowing in answering questions in the embellishing graphics condition, but that the age effect was eliminated when the graphics were explanatory. These data suggest that the conceptually related graphics provided some facilitation for older adults’ comprehension, even though it was not sufficient to improve their performance beyond the level seen in the no graphics condition. However, the graphics in this study were presented in the margins of the leaflets, so perhaps reducing the spatial distance between the text and the graphics would increase older adults’ processing of the graphics, thereby enhancing comprehension even further. This prediction, if supported, could have obvious practical benefits for older adults’ everyday reading.

Given that older people seem to benefit more from multisensory integration (Mozolic et al., 2012), they may also find it easier to read written information while concurrently listening to it. Wright et al. (2008) tested this prediction. Participants performed an “open-book” reading test on the computer and had the option of choosing whether or not they would like to simultaneously listen to the information while reading it. The researchers reported that 41% of older participants chose the listening option regularly. There was no difference in test accuracy or speed between listeners and non-listeners, but pre-test group differences in cognitive ability might have obscured any benefit of listening. This study suggests that a sizeable proportion of older adults, especially those with lower cognitive capabilities, may prefer to learn information presented in multiple modalities simultaneously instead of simply reading written text.

The findings reviewed above (e.g., May, 1999; Yang and Hasher, 2007) make it clear that the processing of target items can be influenced by the conceptual relevance of distracter items. Therefore, it may also be possible that distracters can influence the *depth* of target processing. Since the depth of target processing has been shown to influence retention of to-be-remembered items (Craik and Lockhart, 1972; Craik and Tulving, 1975), it may be possible to improve memory in older adults by manipulating the nature of distraction at encoding. For example, when learning a list of words in the presence of distraction, the depth with which to-be-remembered words are processed could conceivably be influenced by the nature of the relationship between the to-be-remembered words and distracter words. If a distracter cued a shallow feature of the to-be-remembered word (e.g., its font), it may facilitate a shallow processing of the word. On the other hand, if the distracter cued a conceptual feature of the to-be-remembered word (e.g., its closest semantic associate) then the word may be processed more deeply, and therefore it may be better remembered.

Although this specific prediction has not been tested, one study to date does support the idea that memory can be improved in older adults through the processing of congruent distraction. In three experiments, Biss et al. (2013) had older and younger adults learn and recall a list of words, followed by a surprise delayed recall test. In the delay before the final recall, participants performed a working memory task in which some of the words from the initial memory task were repeated as distraction. Older adults, but not younger adults, showed reduced forgetting of the words that were repeated as distraction compared to words that did not repeat. Thus, congruent distraction can improve memory by reactivating, or facilitating processing of, target information in older adults.

## POTENTIAL APPLICATIONS

In the following section we offer some speculations about real-world benefits that might result from the presence of congruent distraction in the lives of older people.

### TEACHING AND INSTRUCTION

Learning a new skill and engaging in new activities are among the most effective ways that people can preserve their cognitive functioning in old age (Park et al., 2014). Therefore, it is critical that instructional information intended for an older audience is created in such a way that facilitates optimal understanding. Based on the findings of Griffin and Wright (2009), it seems that instructional materials should be straightforward and free of unnecessary visual clutter, including graphics, unless the distracting information reinforces the concepts being taught.

### MEMORY

Since there is much empirical evidence to suggest that older adults encode the content of distraction (e.g., Bell et al., 2008), and that distraction can strengthen the representation of memory traces (Biss et al., 2013), it is possible that older adults’ memory might actually be improved by the addition of non-target information to their environment, as long as it reinforces the material they wish to remember. For example, if an older individual wished to remember vocabulary words from a foreign language they are learning, they may wish to play a foreign language radio station in the background while they are commuting or doing housework. An older person may attend to the background sounds more than a young person would, and this may serve to implicitly strengthen their memory of the foreign word meanings they wish to remember.

### DRIVING

Age-related slowing of response time is one of the major safety concerns for drivers over 65 years of age (Anstey et al., 2005). However, older adults’ response times have been shown to be faster than those of young adults when the target is presented in multiple modalities at the same time (Peiffer et al., 2007). Therefore, it is possible that the addition of an automated in-vehicle system that delivers multisensory collision avoidance signals, such as the one proposed by Ho et al. (2007), may be especially beneficial for older drivers. Additionally, the presence of environmental support cues, such as a colored light in the side mirror indicating the safety of a lane change, may provide implicit guidance for older adults’ decision making and serve to prevent accidents. However, in-vehicle assistance systems designed for older drivers need to be created to reduce the amount of irrelevant distraction, not increase it. Systems that require extensive interaction with the driver or provide information that is not of direct relevance, however, well-intentioned, may actually impair the performance of older adults who are more susceptible than young adults to off-topic distraction (Young and Regan, 2007).

## CONCLUSION

The evidence reviewed in this paper suggests that distraction is a double-edged sword for older adults; it can disrupt cognitive performance when incongruent with the task at hand, but it can facilitate performance when congruent. In other words, the notion that all distraction is disruptive is not necessarily true for older adults, who are able to pick up on helpful distraction and use it to their advantage in a way that younger adults do not. Therefore, if one’s goal is to modify environmental conditions so as to optimize cognitive performance, then one should consider age as well as distracter congruence in this process.

However, it is also worth noting that older adults differ widely in their ability to inhibit irrelevant information (Healey et al., 2013), and thus may differ in their ability to use relevant distraction to their advantage. There has been some suggestion in the literature that older individuals with high working memory scores are better at suppressing irrelevant information than are individuals with low working memory scores (Gazzaley et al., 2005; Healey et al., 2013), so perhaps older individuals with impaired working memory would experience the greatest benefit from congruent distraction. There is also some evidence that older adults may have an intuitive sense about whether or not they would benefit from the presence of congruent distraction (Wright et al., 2008), so perhaps the best option is to provide a choice to older individuals so that they can perform in the way that feels most comfortable to them.

## Conflict of Interest Statement

The authors declare that the research was conducted in the absence of any commercial or financial relationships that could be construed as a potential conflict of interest.
